# Clinical outcomes in stump-preserving versus stump-sacrificing anterior cruciate ligament reconstruction; a randomized controlled study

**DOI:** 10.1186/s12891-022-05665-3

**Published:** 2022-07-23

**Authors:** Mahmoud Ahmed El-Desouky, Mostafa Ezzat, Begad Hesham Abdelrazek

**Affiliations:** Department of Trauma and Orthopedics, Faculty of Medicine (Kasr Alainy), Cairo University, Cairo, Egypt

**Keywords:** Anterior cruciate ligament reconstruction, Tibial stump, Stump preservation, Knee proprioception

## Abstract

**Background:**

Anterior Cruciate ligament (ACL) reconstruction (ACLR) aims to restore the anatomy and function of the knee. Although stump preservation during ACLR could be technically challenging, it may improve the revascularization and proprioceptive function of the graft. In this study, we aimed to compare the functional outcome after ACLR with and without stump preservation.

**Methods:**

One hundred and twenty patients with acutely torn ACL and with intact tibial stump were included in this study. Half of them (60 cases) underwent ACLR with stump preservation. The other half (60 cases) had ACLR after total resection of the tibial stump. One hundred and nine out of 120 cases completed their 2 year-follow-up period. All patients were assessed by Tegner activity, Lysholm, and objective International Knee Documentation Committee (IKDC) scores. The side-to-side difference regarding stability was assessed by KT-1000 instrumented Lachman and proprioceptive function was measured by Passive angle reproduction test.

**Results:**

There was no statistically significant difference between both groups regarding Tegner activity, Lysholm, and IKDC scores. Knee stability measured by KT-1000 and complication rate also showed no significant difference. But there was a significant difference in proprioception favoring stump preservation. On the other hand, the operative time was significantly shorter with stump resection. There was no significant difference in the complications rate between both groups and there were no cases with stiffness in either group.

**Conclusion:**

Stump preservation ACLR is a safe technique that yields equivalent functional outcomes to standard ACLR. However; it provides better proprioception. It is more technically challenging, but in experienced hands; it is easily reproducible.

**Trial registration:**

Registration number: NCT05364398. 06/05/2022.

## Introduction

Anterior cruciate ligament (ACL) injury is the most common knee ligamentous injury. It has a major role in resisting abnormal anterior translation and anterolateral rotation of the tibia on the femur. In addition to its function as a knee stabilizer, it also has proprioceptive functions [1, 2].

Anterior cruciate ligament reconstruction (ACLR) is the standard treatment to restore anatomy and function. With improved surgical and arthroscopy instrumentation and technology, the outcome of ACLR is variable. It depends on several varying factors such as graft thickness, fixation method, and tunnel position [2, 3].

Despite how rapidly the technique of ACLR evolved; the success rate is still around 80–90% as reported in some studies, with graft failures reported in up to 8% [4, 5]. Numerous studies addressed the surgical technique as a cause of failure and propose modifications that might improve results. Fewer studies discussed graft incorporation as a cause of failure (biological failure) that can be significantly improved by ACL remnant preservation [6–8].

Studies on the microscopic anatomy of the ACL stump identified the presence of mechanoreceptors and blood vessels that may persist for up to 3 years following injury [9]. Preservation of this stump is believed to enhance cellular proliferation, graft synovial coverage, and neovascularization, all of which subsequently promote proprioceptive recovery [9–12]. Therefore, stump preservation may lead to a lower risk of failure and subsequent revision surgeries [13].

Stump preservation during ACLR was first described by Adachi et al., more than two decades ago [14]. Although it is more technically demanding and prolongs the operative time, the proprioceptive function of ACL and its restoration after reconstruction has been studied clinically, radiologically, and histologically [7, 15, 16].

Our hypothesis is that stump-preserving ACLR can lead to superior clinical outcomes to stump-sacrificing ACLR. In this study, we aimed to compare both techniques regarding proprioception, functional scores, anteroposterior knee stability, and complications rate.

## Methods

From April 2017 to April 2020 one hundred and twenty patients with recently torn ACL (< 6 months) suitable for ACLR were enrolled in this study after exclusion of cases with multi-ligamentous injury, failed previous ACLR, associated mal-alignments, and cases with no identifiable stump.

This was a prospective randomized controlled study with parallel-arm design that was conducted in our department after obtaining the approval of the Research Ethics Committee (REC) with the approval number MD-09–2017. Patients enrolled in the study were recruited from those attending the outpatient clinic at our institute after being evaluated by one of the investigators.

**Fig. 1:**
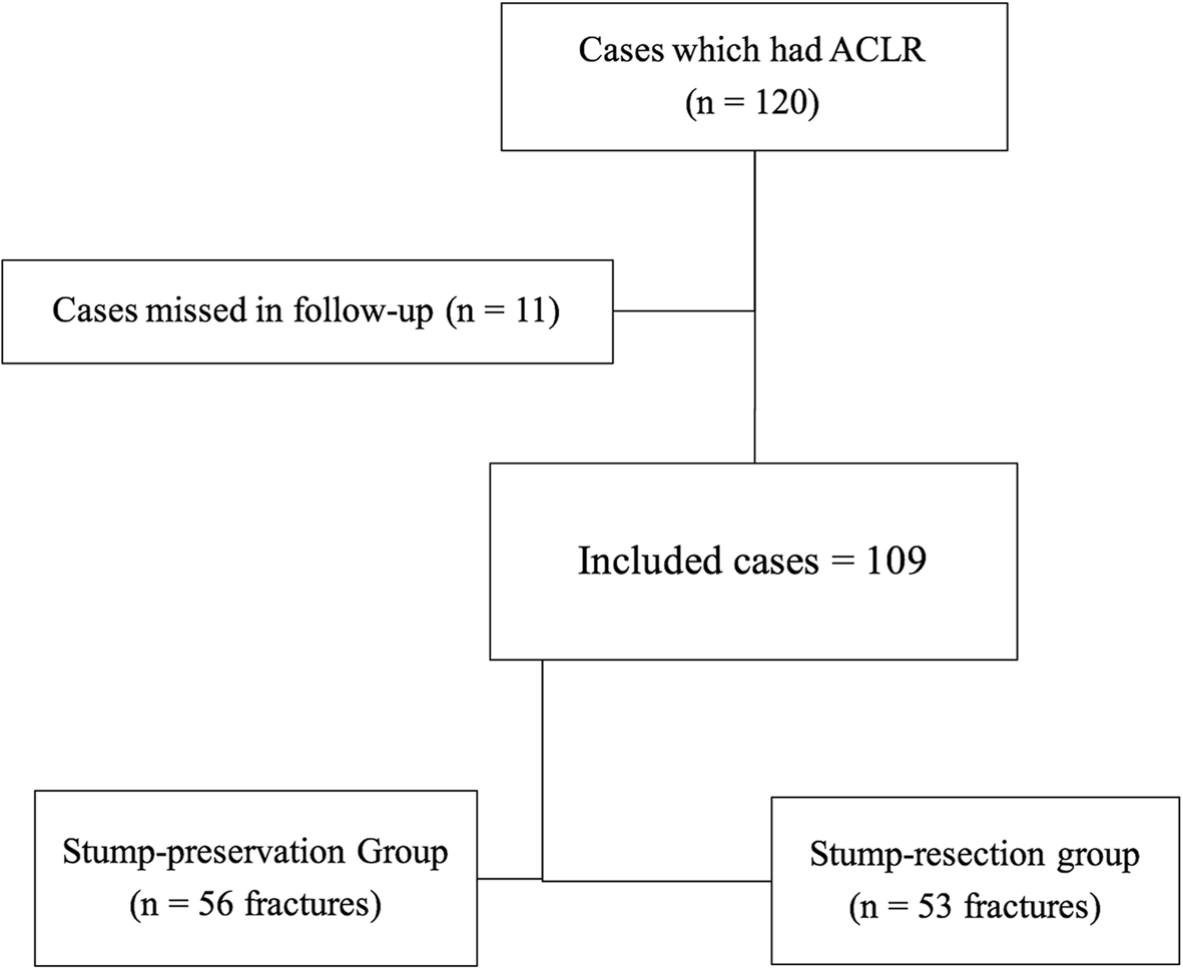
Flow chart of the included cases

We used block randomization technique to generate equal groups. Concealment was done by the closed envelope technique. Cards with numbers from 1 to 120 were put in 120 closed envelopes. Each time one of them was chosen by the operating surgeon on the same day of surgery. Included patients were allocated to two groups by the even/odd numbers technique. Group A (odd numbers) included 60 patients who had ACLR with stump preservation, and group B (even numbers) included 60 patients who had ACLR after stump resection. A total of 11 patients were lost to follow-up, leaving 109 patients at the end of the study who completed 2 years of follow-up (Fig. 1).

The primary outcomes of this trial included the evaluation of both knee stability and proprioceptive functions. While the secondary outcomes included evaluation of the functional outcome through Tegner activity, Lysholm, and the IKDC functional scores.

Before surgery, all patients were subjected to thorough clinical evaluation and magnetic resonance imaging (MRI) of the knee to confirm the presence of ACL injury and to detect any other associated ligamentous, or meniscal injuries. Patients were preoperatively evaluated using Tegner activity, and Lysholm scores, and by the IKDC examination form [17–19].

### Surgical procedure

All patients were operated upon in the supine position under spinal anesthesia and pneumatic tourniquet control. Examination under anesthesia was carried out to exclude any multi-ligamentous injuries.

Hamstring autograft was used in all patients. Diagnostic knee arthroscopy was carried out using standard anterolateral, anteromedial, and accessory anteromedial portals. Examination of the ACL and any meniscal or articular cartilage pathology was performed. In 15 out of 48 patients with associated meniscal injuries, meniscal repair was done using either Fast-Fix device (Smith & Nephew), or the outside-in suture technique. Partial meniscectomy was performed in the other 33 cases. The choice between these modalities was an intraoperative decision made by the surgeon depending on patients’ factors and meniscal tear factors including age, the pattern of the tear, and the site of the tear.

Preparation of the intercondylar notch was performed using an arthroscopic shaver. Preservation of tibial stump fibers was done in (Group A = Stump-preservation group) while both femoral and tibial stumps were debrided in (Group B = Stump-resection group).

**Fig. 2:**
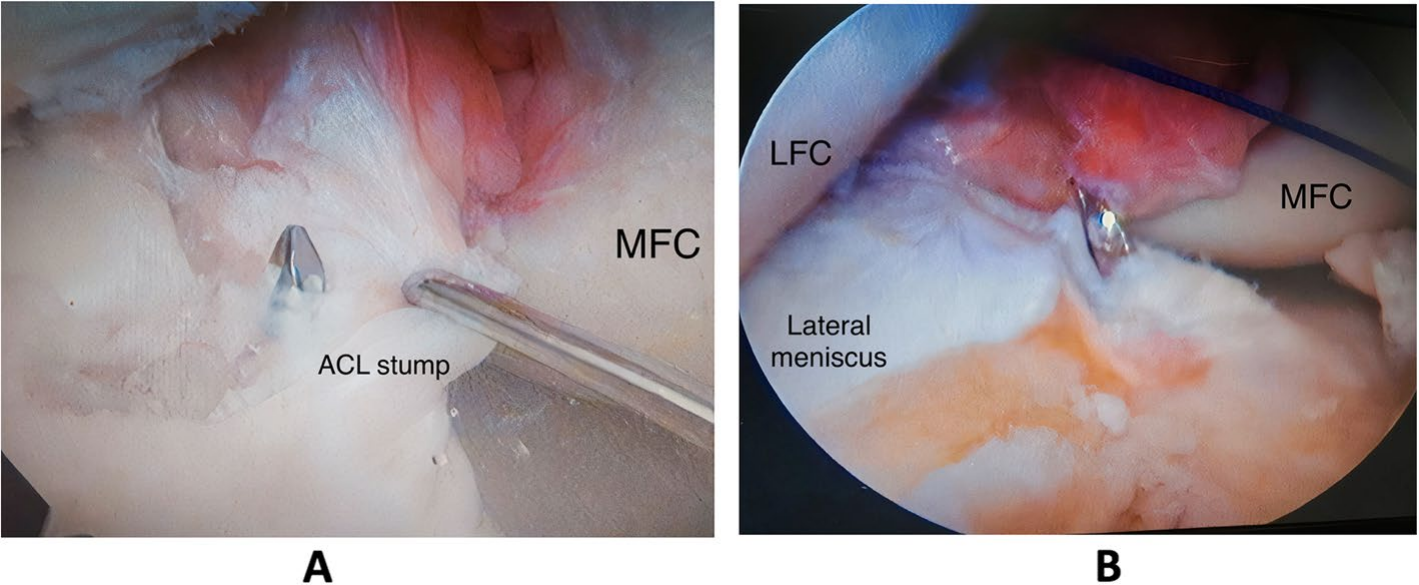
**A** Arthroscopic view of the right knee showing the guidewire tip at the tibial stump in a case in the stump-preservation group, **B** Arthroscopic view of the right knee showing the guidewire tip at the tibial footprint in a case in the stump-resection group (MFC; Medial femoral condyle, LFC; Lateral femoral condyle)

Anatomic drilling of the femoral followed by the tibial tunnel was performed. While the knee flexed 120° the femoral insertion was marked at a point located approximately at 40% of the proximal to distal distance of the lateral notch and is centered between the lateral intercondylar ridge and the posterior edge of the lateral femoral condyle. Then a guide pin was introduced before the tunnel was drilled using a proper size reamer according to the diameter of the proximal part of the graft with a depth of 30 – 35 mm. In group (A) patients the tibial tunnel was aimed at the middle of the tibial stump. While in group (B) patients in whom the stump was previously resected it was aimed at the level of the posterior border of the anterior horn of the lateral meniscus, seven mm anterior to the posterior cruciate ligament (PCL) (Fig. 2).

The graft was passed through tunnels and then fixed at the femoral side using either BIORCI Interference Screw (Smith & Nephew), Mitek Interference Screw (DePuy Synthes), or TightRope (Arthrex). It was fixed at the tibial side using either BIORCI Interference Screw (Smith & Nephew), or Mitek Interference Screw (DePuy Synthes) (Table 1).

**Table 1:** Demographic features of the included cases, methods of fixation, and Pre. and post-operative Lysholm scores

			**Total** **(*n* = 109)**		**Group (A)** **Stump-preservation group** **(*n* = 56)**		**Group (B)** **Stump-resection group** **(*n* = 53)**		***P*-value**
			**Count**	**%**	**Count**	**%**	**Count**	**%**	
**Sex**	**Male**		104	95.4%	52	92.9%	52	98.1%	1
	**Female**		5	4.6%	4	7.1%	1	0.9%	
**Side**	**Right**		66	60.6%	39	69.6%	27	50.9%	0.184
	**Left**		43	39.4%	17	30.4%	26	49.1%	
**Mode of injury**	**Non-contact injury**		88	80.7%	42	75.0%	46	86.8%	0.166
	**Contact Injury**		21	19.3%	14	25.0%	7	13.2%	
**Meniscal injury**	**Medial**		31	28.4%	16	28.6%	15	28.3%	0.759
	**Lateral**		16	14.7%	10	17.8%	6	11.3%	
	**Medial & Lateral**		1	0.9%	1	1.8%	0	0.0%	
	**None**		61	56.0%	29	51.8%	32	60.4%	
**Meniscal procedure**	**Repair**		15	31.3%	8	29.6%	7	33.3%	0.860
	**Partial meniscectomy**		33	68.7%	19	70.4%	14	66.7%	
**Femoral fixation**	**TightRope (Arthrex)**		29	26.6%	11	19.6%	18	34.0%	0.293
	**Interference screw (Smith & Nephew)**		58	53.2%	35	62.5%	23	43.4%	
	**Interference screw (Depuy Synthes)**		22	20.2%	10	17.9%	12	22.6%	
**Tibial fixation**	**Interference screw (Smith & Nephew)**		87	79.8%	47	83.9%	40	75.5%	0.519
	**Interference screw (Depuy Synthes)**		22	20.2%	9	16.1%	13	24.5%	
**Lysholm Score**	**Preoperative**	**Excellent**	0	0.0%	0	0.0%	0	0.0%	0.412
		**Good**	3	2.8%	3	5.4%	0	0.0%	
		**Fair**	33	30.3%	20	35.7%	13	24.5%	
		**Poor**	73	66.9%	33	58.9%	40	75.5%	
	**Postoperative**	**Excellent**	40	36.7%	21	37.5%	19	35.9%	0.720
		**Good**	33	30.3%	19	33.9%	14	26.4%	
		**Fair**	35	32.1%	15	26.8%	20	37.7%	
		**Poor**	1	0.9%	1	1.8%	0	0.0%	
	***P*- value** **(Pre. vs. Postoperative)**		< 0.001		< 0.001		< 0.001		

**Fig. 3:**
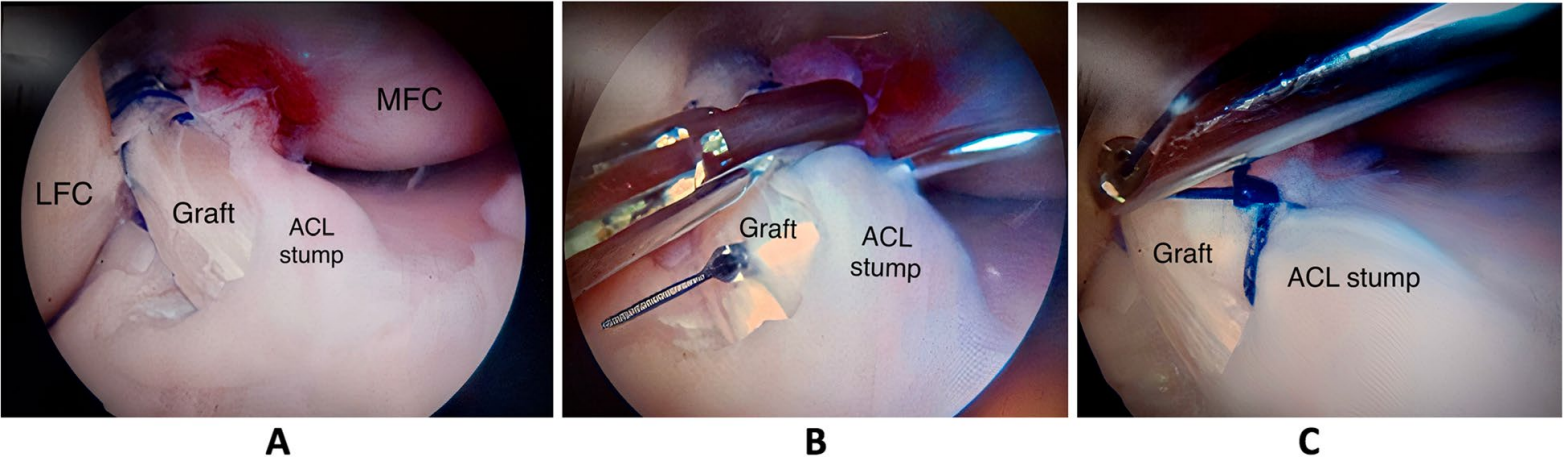
Arthroscopic view of the right knee showing the tibial stump while it is sutured to the ACL graft in stump-preservation technique, (MFC; Medial femoral condyle, LFC; Lateral femoral condyle)

**Fig.4:**
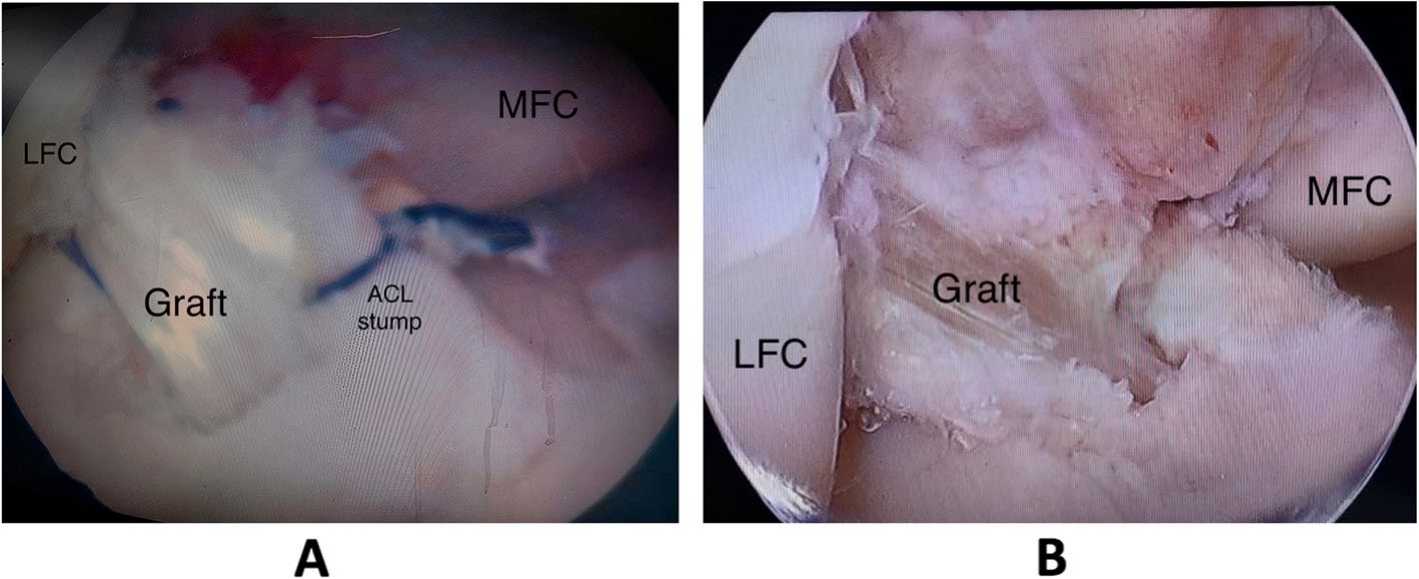
**A** Arthroscopic view of the right knee showing reconstructed ACL in the stump-preservation technique, **B** Arthroscopic view of the right knee showing reconstructed ACL in the stump-resection technique (MFC; Medial femoral condyle, LFC; Lateral femoral condyle)

In group (A), a grasper was used for tensioning the stump while suturing it to the graft with a No. 1 Prolene suture, then tied to it (Figs. 3 and 4). Finally, the absence of graft impingement was checked arthroscopically in extension. Then a drain was used and closure of the arthroscopy portals and graft harvesting wound was done. The wounds were covered with sterile adhesive plasters and a crepe bandage. Postoperative radiographs were obtained routinely for all patients to assess tunnel positioning.

### Postoperative regimen

**Fig. 5:**
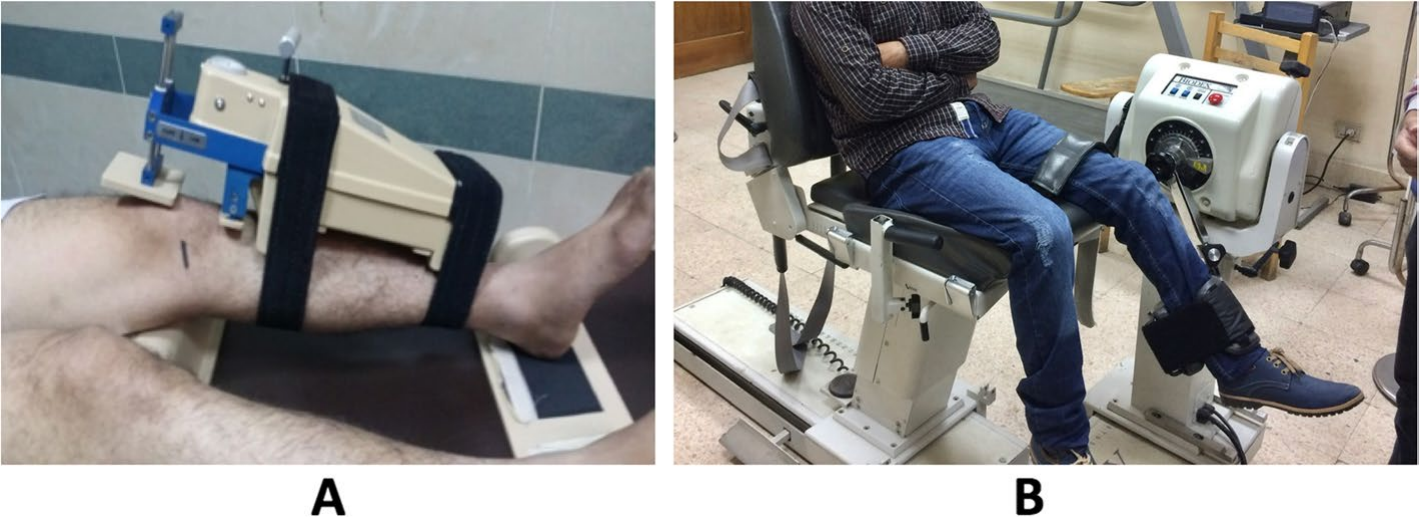
**A** Instrumented Lachman examination using KT-1000 Arthrometer, **B** Proprioception measurement by Passive angle reproduction test

Patients were discharged on the second day after surgery after drain removal. They were encouraged to achieve early full active knee extension and weight-bearing was allowed as tolerated. Stitches removal was done after two weeks. Then patients were followed up every two weeks in the first 12 weeks after surgery during which gradual restoration of full knee range of motion, progression to full weight-bearing, and muscle strengthening were instructed through a rehabilitation program. Running was allowed after 3 months and participation in contact sports after 6 to 9 months. In patients who had meniscal repair, partial weight-bearing was allowed in the first 4–6 weeks only while wearing a knee brace locked in full extension. Meanwhile, flexion exercises were restricted to less than 90°. After that gradual restoration of full weight-bearing and full knee flexion was initiated. The average follow-up period was 25.47 months (range 24–29 months). At the last visit, patients’ functional outcome was evaluated using Tegner activity, Lysholm, and IKDC scoring. Also, knee stability was assessed and compared with the sound side using KT-1000 (MEDmetric® Knee Ligament Arthrometer® model KT1000™). The proprioceptive function was evaluated by the Passive angle reproduction test (Biodex System Pro 3) done at 30° of knee flexion and compared to the opposite side (Fig. 5) [20].

The preoperative evaluation was performed by one of the investigators before allocating the type of surgery done to the patient. While postoperative evaluation and measurements were performed by the second author who was together with the physiotherapists and the data analysts blinded to the type of the surgery.

### Statistical methods

The sample size of each group was calculated on G-power program version 3.1.9.7. Sample sizes of 50 patients in each group were calculated to yield 80% statistical power.

Data were coded and entered using the statistical package for the Social Sciences (SPSS) version 28 (IBM Corp., Armonk, NY, USA). Data were summarized using mean and standard deviation, minimum and maximum for quantitative variables and frequencies (number of cases), and relative frequencies (percentages) for categorical variables. Comparisons between groups were done using unpaired t-test in normally distributed quantitative variables while non-parametric Mann–Whitney test was used for non-normally distributed quantitative variables. Comparison between values measured pre and post in the same patient was done using paired t-test in numerical data and marginal homogeneity test in ordinal data (Chan, 2003a). For comparing categorical data, Chi-square (χ2) test was performed. Exact test was used instead when the expected frequency is less than 5 (Chan, 2003b). *P*-values less than 0.05 were considered as statistically significant.

## Results

The mean age was 27.7 ± 7.2 years (ranging from 18 – 41 years old). There were 104 males (95.4%) and 5 females (5.6%). The average body mass index was 24.4 ± 3.5 kg/m^2^. Eighty-eight out of 109 patients (80.7%) had a non-contact injury during athletic activity. The other 21 cases (19.3%) were injured due to contact injury. Forty-eight cases (44.0%) had associated meniscal injuries. There was no statistically significant difference between both groups in demographic characteristics (Table 1).

The time interval between injury and intervention was comparable in both groups and it ranged from 5 to 24 weeks with a mean value of 15.7 weeks. The operative time ranged from 43 to 146 min with a mean value of 89.0 min. The mean operative time in the stump preservation group was significantly longer (106.8 ± 24.1 min) as compared to the stump resection group (71.2 ± 21.2 min) (*P* < 0.001) (Table 2).

**Table 2:** Comparison of operative data and results in both groups

		**Total**		**Group (A)** **Stump-preservation group**		**Group (B)** **Stump-resection group**		***P*-value**
		**Mean**	**SD**	**Mean**	**SD**	**Mean**	**SD**	
**Time to surgery (weeks)**		15.7	5.6	16.3	5.7	15.2	5.6	0.453
**Operative time (minutes)**		89.0	28.8	106.8	24.1	71.2	21.2	< 0.001
**Tegner activity score**	**Pre-injury**	6.3	1.1	6.3	1.1	6.2	1.1	0.550
	**Postoperative**	5.8	0.9	5.9	0.9	5.7	1.0	0.286
	***P*- value (Pre-injury vs. Postoperative)**	< 0.001		0.003		0.003		
**Side-to side difference in KT-1000**		1.2	1.1	1.2	1.1	1.2	1.1	0.805
**Absolute proprioception error**		2.6	0.9	2.3	0.9	2.9	0.5	0.038

**Fig. 6:**
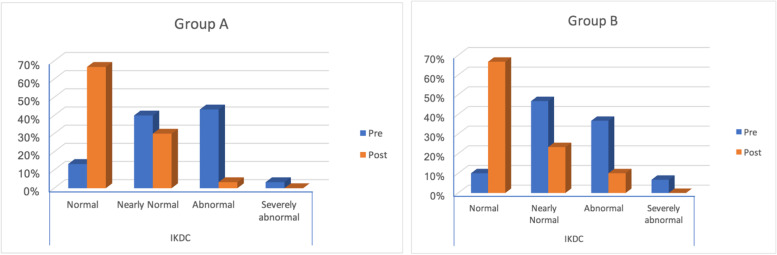
IKDC scoring preoperatively and postoperatively in group (**A**) and group (**B**)

There were no significant differences between both groups regarding postoperative Tegner activity, Lysholm, or IKDC scores (*P* = 0.286, 0.720, 0.643 respectively) (Table 1). Regarding IKDC scores, in group (A) 37 patients (66.1%) postoperatively had normal results and 18 patients (32.1%) had nearly normal results compared to 36 patients (67.9%) and 13 patients (24.5%) respectively in group (B) (Fig. 6).

There was no statistically significant difference between both groups in terms of knee stability calculated by the side-to-side difference in the KT-1000 assessment (*P *= 0.805). While absolute proprioception error recorded while performing the Passive angle reproduction test measured at 30° knee flexion was 2.3 ± 0.9 in group (A) and 2.9 ± 0.5 in group (B) indicating a significant difference (*P* = 0.038) (Table 2).

One patient in the stump preservation group (1.8%) developed postoperative infection which was managed by arthroscopic debridement and antibiotics course and subsided after two weeks. Five patients (4.6%) had postoperative hematoma; three patients were in the stump preservation and 2 in the stump resection group. It appeared in the first 2 weeks and was managed by cryotherapy and stoppage of prophylactic anticoagulants course. Six patients; three in each group had quadriceps wasting > 2 cm compared to the opposite side and the patients experienced a persistent feeling of giving way. There was a single case of deep venous thrombosis (DVT) in the stump resection group (1.9%) which was detected in the first two weeks after surgery and was managed by rest, and LMW heparin subcutaneous injection for two weeks followed by oral warfarin. There were no cases with stiffness (loss of > 5° extension or having flexion range < 120°) in either group. Until the last follow-up visit, none of the patients experienced ACL failure. Generally, there was no significant difference in the complication rate between both groups.

## Discussion

The tibial stump of torn ACL is of particular interest during ACLR as it is believed to provide blood supply and synovial coverage which optimize and accelerate graft healing. Furthermore, it is rich in mechanoreceptors, therefore; providing better proprioception, quicker rehabilitation, and return to sports [9–12].

In this series, we compared the results of 56 patients who underwent stump-preservation ACLR to 53 patients with stump-resection ACLR. Although relatively better functional outcome Tegner activity, Lysholm, or IKDC scores were obtained in the stump-preservation group, the results did not achieve a statistically significant difference (*P* = 0.286, 0.720, 0.643 respectively). The mean operative time in the stump-preservation group was significantly longer than it in the stump resection group (*p* < 0.001). KT-1000 assessments revealed no statistically significant difference between both groups (*P* = 0.805). Meanwhile, the proprioceptive function assessed by the Passive angle reproduction test was significantly better in the stump preservation group (*P* = 0.038). There was no significant difference in the complication rate between both groups.

In the study of Gupta T.P. et al., a significantly higher rate of graft re-rupture was seen in the group who underwent a remnant-sacrificing procedure. The KT-2000 instrumented Lachman also showed a significantly greater side-to-side difference favoring the remnant-preservation group. This is different from our findings, as we found no significant side-to-side difference between both groups regarding stability. Their study revealed no statistically significant difference in the postoperative Lysholm and Tegner activity scores between both groups which was consistent with the findings of the present series [21].

In the study of Naroka et al., on 151 consecutive ACLR using the anatomic double-bundle (ADB) technique, they found that remnant preservation during ACLR did not improve knee stability and outcomes at two years, nor did it accelerate graft incorporation, when compared to standard ACLR. The reason might be that in their series they used ADB ACLR which is expected to provide better stability for both groups. Regarding operative time, they reported no significant difference between both groups. In the present study, however; operative time was significantly longer for the stump preservation group. Again, we owe this contrast to the fact that they used ADB ACLR in all cases which in itself prolongs the operative time. Furthermore, performing ADB reconstruction without debridement of the stump might be very difficult and challenging [22].

In the study of Liu Yufeng et al., on 46 knees they compared ACLR with larger stump (> 1/3 remnant present) and smaller stump (< 1/3 remnant present). They found no statistically significant difference between both groups regarding the Lysholm score and KT-2000 measurements. But there was a significantly better postoperative Tegner activity score and proprioceptive function with the larger stumps. The proprioceptive assessment was done by the reproduction of passive positioning (RPP) and threshold detection of passive motion (TPM) in 15° flexion only 6 months postoperatively compared to the present study which was at 30° after 2 years suggesting better judgment. However, their findings are still consistent with the findings of this series, as there was a significant difference between both groups favoring the stump preservation group [23].

Remnant preservation, however; has its disadvantages. Over being an added step that prolongs the surgical time, it increases the risk of oversized bulky graft or cyclops lesions formation, both of which might lead to notch impingement and loss of full extension [15, 24, 25]. While we did not encounter loss of knee extension in our study, Nakayama et al. reported a higher rate of extension loss in the remnant preservation group; six patients versus only three in the standard ACLR group. This is different from the study of Kondo et al., who reported no difference between both groups in the development of cyclops lesions; nine in the remnant preservation group versus eight in the remnant sacrificing group. They, however; did not report on loss of extension, which reflects the importance of the size of cyclops lesions to predict whether they are significant or not [26, 27].

Another disadvantage is that trying to preserve the tibial stump was reported to increase the margin of error in tibial tunnel placement [28, 29]. However, de Padua et al. who used postoperative 3D-CT to assess the effect of the stump preservation technique on the rate of tibial tunnel malposition reported no significant difference to stump resection [30].

While the present research relied on objective analysis using the objective IKDC score and the Biodex proprioceptive analysis, it lacks a second look arthroscopy or MRI to assess graft incorporation and possible development of cyclops lesions. It also lacks radiographic assessment of the effect of stump preservation on tunnel widening which has been analyzed in several studies depending on the fact that the leakage of synovial fluid through tibial tunnels with stump resection may start the osteolytic process [31–33]. Also, postoperative assessment of tunnel position would be better assessed accurately using computed tomography (CT) rather than X-rays. Another weakness of the present study is that the effect of stump preservation on time to return to preoperative activity was not analyzed. Although we relied on Tegner activity score to assess the level of activity at the final follow-up; time to return to this activity is equally important.

Although we believe that the ideal randomized controlled trials that offer the best quality of evidence should limit all confounding variables, patients with and without associated meniscal injuries were included in this study with variable methods of management including partial meniscectomy or meniscal repair using either Fast-Fix devices or outside-in suture technique. The choice of the proper method of management was made according to what was intraoperatively seen as the adequate treatment at time of surgery depending on patients’ factors and meniscal tear factors including age, the pattern of the tear, and the site of the tear. We tried to stick to one fixation method on the tibial side (interference bioscrew) and 2 methods of fixation on the femoral side (interference bioscrew and cortical button) to limit the external variables as much as we can. The reason we used different manufacturers was according to the availability of the implants in our institute. However, there were no statistically significant differences between both groups regarding femoral and tibial fixation methods (*p* = 0.293 and 0.519 respectively), the incidence of associated meniscal injuries (48.2% in the Stump-preservation group versus 39.6% in the Stump-resection group, *p* = 0.759), or the type of management to the meniscal injury (*p* = 0.860). Therefore, we believe that all these variables will pose very little bias or effect on the clinical outcomes and it shall not be statistically significant.

## Conclusion

Stump preservation ACLR is a safe technique that yields equivalent functional outcomes to standard ACLR. However; it provides better proprioception. It is more technically challenging, but in the experienced hands; it is easily reproducible. It is advised to do ACLR with stump preservation as long as there is an adequate stump and it is not difficult to clearly visualize the tibial footprint.

## Data Availability

The datasets used in the current study are available from the corresponding author upon reasonable request.

## Abbreviations

ACL: Anterior Cruciate Ligament
ACLR: Anterior Cruciate Ligament Reconstruction
IKDC: International Knee Documentation Committee
MRI: Magnetic Resonance Imaging
PCL: Posterior Cruciate Ligament
DVT: Deep Venous Thrombosis
LMW: Low Molecular Weight
ADB: Anatomic Double-bundle
RPP: Reproduction of Passive Positioning
TPM: Threshold detection of Passive Motion
CT: Computed tomography

## References

[1] Duthon VB, Barea C, Abrassart S, Fasel JH, Fritschy D, Ménétrey J (2006). Anatomy of the anterior cruciate ligament. Knee Surg Sports Traumatol Arthrosc.

[2] Markatos K, Kaseta MK, Lallos SN, Korres DS, Efstathopoulos N (2013). The anatomy of the ACL and its importance in ACL reconstruction. Eur J Orthop Surg Traumatol.

[3] Samitier G, Marcano AI, Alentorn-Geli E, Cugat R, Farmer KW, Moser MW (2015). Failure of Anterior Cruciate Ligament Reconstruction. Arch Bone Jt Surg.

[4] Kocher MS, Steadman JR, Briggs K, Zurakowski D, Sterett WI, Hawkins RJ (2002). Determinants of patient satisfaction with outcome after anterior cruciate ligament reconstruction. J Bone Joint Surg Am.

[5] Johnson DL, Coen MJ (1995). Revision ACL surgery. Etiology, indications, techniques, and results. Am J Knee Surg.

[6] Junkin DM, Johnson DL (2008). ACL tibial remnant, to save or not?. Orthopedics.

[7] Lee BI, Kwon SW, Kim JB, Choi HS, Min KD (2008). Comparison of clinical results according to amount of preserved remnant in arthroscopic anterior cruciate ligament reconstruction using quadrupled hamstring graft. Arthroscopy.

[8] Ahn JH, Lee YS, Ha HC (2009). Anterior cruciate ligament reconstruction with preservation of remnant bundle using hamstring autograft: technical note. Arch Orthop Trauma Surg.

[9] Sha L, Xie G, Zhao S, Zhao J (2017). A morphologic and quantitative comparison of mechanoreceptors in the tibial remnants of the ruptured human anterior cruciate ligament. Medicine (Baltimore).

[10] Vavken P, Murray MM (2011). The potential for primary repair of the ACL. Sports Med Arthrosc Rev.

[11] Dhillon MS, Bali K, Vasistha RK (2010). Immunohistological evaluation of proprioceptive potential of the residual stump of injured anterior cruciate ligaments (ACL). Int Orthop.

[12] Cho E, Chen J, Xu C, Zhao J (2022). Remnant preservation may improve proprioception after anterior cruciate ligament reconstruction. J Orthop Traumatol.

[13] Benjamin BR, Eiji K, Rainer S, Joon HW, Kyoung HY, Freddie HF (2020). Anterior cruciate ligament reconstruction with remnant preservation: current concepts. J ISAKOS.

[14] Adachi N, Ochi M, Uchio Y, Sumen Y (2000). Anterior cruciate ligament augmentation under arthroscopy. A minimum 2-year follow-up in 40 patients. Arch Orthop Trauma Surg.

[15] Reda W, Khedr A (2017). Stump Incorporation for Anterior Cruciate Ligament Reconstruction: A Step Towards a More Anatomical Reconstruction. Arthrosc Tech.

[16] Papalia R, Franceschi F, Vasta S, Di Martino A, Maffulli N, Denaro V (2012). Sparing the anterior cruciate ligament remnant: is it worth the hassle?. Br Med Bull.

[17] Tegner Y, Lysholm J (1985). Rating systems in the evaluation of knee ligament injuries. Clin Orthop Relat Res.

[18] Lysholm J, Gillquist J (1982). Evaluation of knee ligament surgery results with special emphasis on use of a scoring scale. Am J Sports Med.

[19] Rossi MJ, Lubowitz JH, Guttmann D (2002). Development and validation of the International Knee Documentation Committee Subjective Knee Form. Am J Sports Med.

[20] Fridén T, Roberts D, Zätterström R, Lindstrand A, Moritz U (1996). Proprioception in the nearly extended knee. Measurements of position and movement in healthy individuals and in symptomatic anterior cruciate ligament injured patients. Knee Surg Sports Traumatol Arthrosc.

[21] Chan YH. Biostatistics 102: quantitative data--parametric & non-parametric tests. Singapore Med J. 2003;44(8):391–396. 14700417

[22] Chan YH (2003). Biostatistics 103: qualitative data - tests of independence. Singapore Med J.

[23] Liu Y, Li C, Ma N (2022). Proprioceptive and Clinical Outcomes after Remnant Preserved Anterior Cruciate Ligament Reconstruction: Assessment with Minimal Confounding Factors. Orthop Surg.

[24] Tonin M, Saciri V, Veselko M, Rotter A (2001). Progressive loss of knee extension after injury: cyclops syndrome due to a lesion of the anterior cruciate ligament. Am J Sports Med.

[25] Cha J, Choi SH, Kwon JW, Lee SH, Ahn JH (2012). Analysis of cyclops lesions after different anterior cruciate ligament reconstructions: a comparison of the single-bundle and remnant bundle preservation techniques. Skeletal Radiol.

[26] Nakayama H, Kambara S, Iseki T, Kanto R, Kurosaka K, Yoshiya S (2017). Double-bundle anterior cruciate ligament reconstruction with and without remnant preservation - Comparison of early postoperative outcomes and complications. Knee.

[27] Kondo E, Yasuda K, Onodera J, Kawaguchi Y, Kitamura N (2015). Effects of Remnant Tissue Preservation on Clinical and Arthroscopic Results After Anatomic Double-Bundle Anterior Cruciate Ligament Reconstruction. Am J Sports Med.

[28] Webster KE, Murgier J, Feller JA, Klemm HJ, Devitt BM, Whitehead TS (2021). Preservation of the Tibial Stump During Anterior Cruciate Ligament Reconstruction Surgery Did Not Increase the Rate of Surgery for Symptomatic Cyclops Lesions. Orthop J Sports Med.

[29] Ahn JH, Wang JH, Lee YS, Kim JG, Kang JH, Koh KH (2011). Anterior cruciate ligament reconstruction using remnant preservation and a femoral tensioning technique: clinical and magnetic resonance imaging results. Arthroscopy.

[30] de Padua VBC, Saithna A, Chagas EFB (2021). Rate of Tibial Tunnel Malposition Is Not Changed by Drilling Entirely Within the Stump of Preserved Remnants During ACL Reconstruction: A Prospective Comparative 3D-CT Study. Orthop J Sports Med.

[31] Demirağ B, Ermutlu C, Aydemir F, Durak K (2012). A comparison of clinical outcome of augmentation and standard reconstruction techniques for partial anterior cruciate ligament tears. Eklem Hastalik Cerrahisi.

[32] Zhang Q, Zhang S, Cao X, Liu L, Liu Y, Li R (2014). The effect of remnant preservation on tibial tunnel enlargement in ACL reconstruction with hamstring autograft: a prospective randomized controlled trial. Knee Surg Sports Traumatol Arthrosc.

[33] Zysk SP, Fraunberger P, Veihelmann A (2004). Tunnel enlargement and changes in synovial fluid cytokine profile following anterior cruciate ligament reconstruction with patellar tendon and hamstring tendon autografts. Knee Surg Sports Traumatol Arthrosc.

